# Modified immunoscore improves the prediction of progression-free survival in patients with non-muscle-invasive bladder cancer: A digital pathology study

**DOI:** 10.3389/fonc.2022.964672

**Published:** 2022-09-23

**Authors:** Uwe Bieri, Dominik Enderlin, Lorenz Buser, Marian S. Wettstein, Daniel Eberli, Holger Moch, Thomas Hermanns, Cédric Poyet

**Affiliations:** ^1^ Department of Urology, University Hospital of Zurich, University of Zurich, Zurich, Switzerland; ^2^ Department of Pathology and Molecular Pathology, University Hospital of Zurich, University of Zurich, Zurich, Switzerland

**Keywords:** bladder cancer, immunoscore, biomarker, progression, prognosis, digital pathology

## Abstract

Tumour-infiltrating lymphocytes (TIL), known to be of prognostic value in various solid tumours, have been in the focus of research in the last years. TIL are often quantified *via* IMMUNOSCORE ^®^ (IS), a scoring system based on TIL cell densities. Recent studies were able to replicate these findings for muscle-invasive bladder cancer (MIBC), however data regarding non-muscle-invasive bladder cancer (NMIBC) are scarce. This study aimed to evaluate the value of a modified Immunoscore (mIS) as a predictive marker for NMIBC prognosis using tissue-micro-arrays (TMAs). We analysed two TMAs containing 316 samples from 158 patients with NMIBC, stained for CD3, CD8, CD45RO and FOXP3. Stained TIL were captured by digital pathology, cumulated, averaged, and reported as density (stained cells per mm²). The mIS was then constructed based on density of all four immune-cell types. Clinical, pathological and follow-up data were collected retrospectively. Univariable and multivariable cox regression analysis was performed to assess the potential value of mIS as a predictor for progression free survival (PFS) and recurrence-free-survival (RFS). Patients within “European Organisation for Research and Treatment of Cancer” (EORTC) risk groups were further substratified in high mIS and low mIS subgroups. Finally log-rank test was used to compare the different survival curves. The median age in our cohort was 68 years (Interquartile Range (IQR): 60 - 76), and 117 (74%) patients were male. A total of 26 patients (16.5%) were classified as EORTC low risk, 45 (28.5%) as intermediate risk and 87 (55.1%) as high risk. Patients in the EORTC high risk group with low mIS showed a shorter PFS in comparison to high mIS (HR 2.9, CI 0.79 – 11.0, p=0.082). In contrast, no predictive potential regarding PFS was observed in intermediate or low risk groups. Furthermore, mIS was not able to predict RFS in any EORTC risk group. mIS could be utilized to predict prognosis more accurately in high-risk patients with NMIBC by identifying those with higher or lower risk of progression. Therefore, mIS could be used to allocate these highrisk patients to more streamlined follow-up or more aggressive treatment strategies.

## Introduction

Bladder cancer (BC) is the 10th most diagnosed cancer worldwide, while being around four times more common in men than women (1). Around 70% of all BC is diagnosed at a non-muscle-invasive stage (2). Non-muscle-invasive bladder cancer (NMIBC) is usually treated by transurethral endoscopic resection (3), and about 55% of patients experience disease recurrence within 3 years after initial resection (4). Therefore, depending on risk stage, adjuvant local intravesical therapy, either chemotherapeutic agents or more often Bacillus Calmette-Guérin (BCG) is applied (3, 5). Nevertheless, a relevant number of patients fail to respond to this adjuvant treatments and as a result the cancer can recur or even progress to muscle-invasive BC (MIBC) (6, 7). Thus, early more aggressive treatment as for example radical cystectomy (RC) can be discussed for very high-risk NMIBC patients (3, 8).

Clinically the most established tool to predict disease recurrence and progression is the “European Organisation for Research and Treatment of Cancer” (EORTC) risk stratification, based on six predictors, such as tumor number, tumor size, prior recurrence rate, tumor stage and grade as well as the presence of carcinoma *in situ* (CIS) (9). The application of this tool is widely accepted among clinicians and recommended in current European Association of Urology guidelines for NMIBC (3). However, it has limitations, especially in high-risk patients where the model overestimates the risk of disease recurrence and progression (10).

Further tools to more accurately predict prognosis in patients with NMIBC are urgently needed (11, 12). Tumour-infiltrating lymphocytes (TIL) have been shown to be a valuable prognostic marker in several different solid tumours, indicating that immune system activation is associated with delayed cancer progression (12–15). This is especially relevant in NMIBC because BCG is known to enhance the antitumoral effect of T-cells and CD4- and CD8-positive cells are regarded to be essential for tumour elimination in NMIBC undergoing BCG treatment (16–18). A method to quantify TIL is IMMUNOSCORE ^®^ (IS) (19–22), a standardized scoring system based on CD3 and CD8 immune cell densities in the tumour tissue (23). IS is already included in current European Guidelines for Medical Oncology for colorectal cancer to further tailor adjuvant therapeutic strategies in difficult cases (24).

In muscle-invasive BC higher counts of tumour-infiltrating CD3 and CD8 lymphocytes have been linked to favourable disease outcomes (12, 25–27) and even to a higher response rate to neoadjuvant chemotherapy (27). In MIBC, we have recently shown that a higher density of TIL was associated with longer progression-free-survival (PFS) in patients in the American Joint Committee on Cancer stage IIIa (28). However, data for NMIBC are scarce.

The aim of this study was to explore the value of a modified IS (mIS) as a prognostic marker for PFS and recurrence-free-survival (RFS) in NMIBC.

## Methods

### Tissue microarrays

TMA is an established high-throughput technique, which enables the simultaneous assessment of several molecular targets and are often used in tumour research (29). We stained two TMAs containing 316 samples from 158 patients with NMIBC after initial transurethral resection. Papillary urothelial neoplasm of low malignant potential was not considered in this study as current guidelines clearly distinguish it from more aggressive NMIBC (30). Two tissue cores per patient were processed for analysis. The samples have been collected between 1990 and 2006 by the Institute of surgical Pathology of the University Hospital of Zürich. The TMAs have been constructed as previously described (31, 32). Both cohorts were approved by the local Ethics committee (StV-Nr. 25/2008 & 02/2009).

### Immunhistochemistry

The TMAs were immunohistochemically stained with CD3 antibodies for T-cells, CD8 antibodies for cytotoxic T-cells, FOXP3 antibodies for regulatory T-cells (Treg) and CD45RO antibodies for memory T-cells. Then the stained slides were scanned and imported into QuPath (version 0.1.2), a software for digital pathology image analysis (33).

Automated analysis was performed, analogous to our previous study (28), to detect and quantify each immune cell subpopulation as described in the following steps:

1. QuPath’s automated “TMA dearrayer” was used to identify tissue cores. The resulting TMA grid was manually verified and amended where necessary.
2. Stain vector and background estimates were applied to improve stain separation using color deconvolution by QuPath’s “Estimate stain vectors” command.
3. QuPath’s built-in “Simple tissue detection” and “Fast cell counts” commands were applied. The measurements were visually controlled by a board-certified pathologist (Lorenz Buser) and the parameters manually adjusted until convincing results could be achieved, in particular the “thresholdDAB value” determining the cut-off for positive cell count.
4. Output was cumulated, averaged and reported as positive counts (pc), negative (nc) counts, ratio (pc/pc+nc) and density (pc/mm^2).

### Construction of the modified immunoscore prediction model

To explore the potential of the mIS, it was necessary to define the most relevant value from the available source data generated by the above-mentioned immunohistochemistry assays. Based on biological reasoning, the averaged density values of the two tissue cores for counts of CD3, CD8, CD45RO and FoxP3 marked TIL per patient were selected to construct the mIS prediction model. Log-transformed average densities of all 4 TIL were used throughout the analyses to mitigate any undesirable effect of extreme values. Based on that data the cohort was then dichotomized in high mIS/favorable risk” and “low mIS/unfavorable risk” groups using the median as a cut-off point.

### Patient data acquisition

Patient data was collected retrospectively (until December 2020). PFS was defined as time in months from initial transurethral resection until first upstaging event, eg, from pTa to pT1-pT4, or pT1 to pT2-pT4, respectively. Additionally, RFS was defined as time in months from initial transurethral resection until local recurrence within the same tumour stage occurred.

### Statistics

Data was analyzed by the statistic program *R (R Core Team, Vienna, Austria)*. An assessment of numeric behavior and collinearity was performed. Univariable and multivariable cox regression analysis was performed to find the most informative predictors and to assess effect size in comparison to other established prognostic factors (age, gender, multifocality, grade and clinical T-stage) regarding the clinically significant outcome PFS and RFS. For multivariable analysis, we summarized non-Immunoscore predictors, such as carcinoma *in situ* or multifocality, into the EORTC risk group classification. Additionally, performance improvement (concordance index) of mIS and individual mIS components (CD3, CD8, CD45RO, FoxP3) on top of the already established EORTC risk groups classifications was analyzed. Finally, patients within the three different EORTC risk groups were further subdivided in low mIS and high mIS strata. Kaplan-Meier (KM) analysis was performed, and the log-rank test was used to compare the different survival curves. Results with p-value <0.05 were considered statistically significant.

## Results

### Patient characteristics

Tissue of 158 (100%) patients was available for TMA construction and final analysis. The median age in our cohort was 68 years (Interquartile Range (IQR): 60 - 76), and 117 (74%) of patients were male (**Table 1**). Stage distribution was 90 (57%) for pTa and 68 (43%) for pT1. A total of 26 (16.5%) patients were classified as EORTC low risk, 45 (28.5%) as medium and 87 (55.1%) as high risk. Median follow-up time after first transurethral resection was 122.6 months (IQR: 74.5 – 192.6) and 47 patients (29.7% were followed for more than 180 months. During follow-up 86 patients (54.4%) experienced disease recurrence and 23 patients (14.6%) disease progression, 15 patients (9.5%) progressed to muscle-invasive disease.

**Table 1:** Patient characteristics.

Number of patients	158
**Male**	117 (74.1%)
**Female**	41 (25.9%)
**Age (y)**	68 (IQR: 60-76)
**Median follow-up time (m)**	48 (IQR: 74.5–192.6)
**Number of patients with follow-up time:**	
**>60 months**	135 (85.4%)
**>120 months**	80 (50.6%)
**>180 months**	47 (29.7%)
**Primary bladder tumor stage**	
**Ta/Tcis**	90 (57%)
**T1**	68 (43%)
**Primary bladder tumour grade (2004 Classification)**	
**Low grade**	101 (63.9%)
**High grade**	57 (39.1%)
**EORTC risk group**	
**Low risk**	26 (16.5%)
**Medium risk**	45 (28.5%)
**High risk**	87 (55%)
**Number of patients with:**	
**recurrence**	86 (54.4%)
**progression**	23 (14.6%)
**progression to muscle-invasive disease**	15 (9.5%)

Data presented as median (IQR) or number (percent).

IQR, Inter-Quartile-Range; EORTC, European Organisation for Research and Treatment of Cancer.

### Immunohistochemistry

**Figure 1:**
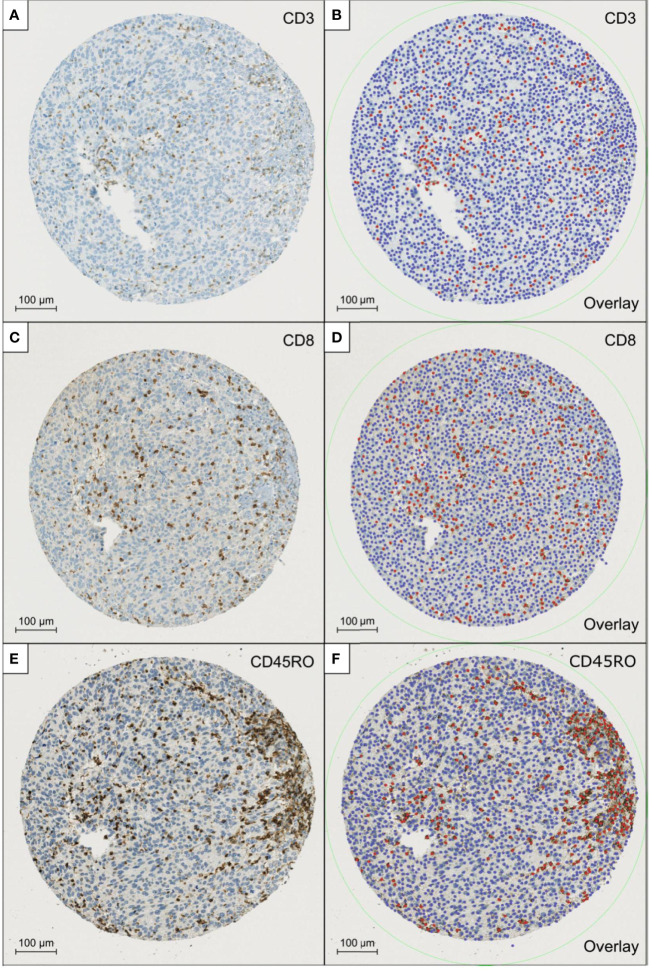
shows a representative example for the scanned TMAs stained with antibodies for CD3 **(A)**, CD8 **(C)**, and CD45RO **(E)** and the corresponding overlays generated by QuPath **(B, D, F)**. Red dots highlight the lymphocytes that where rated positive, while the blue-coloured dots equal the remaining detected cells which were counted as negative.

Immunohistochemical staining of the TMAs was successful in all cases and all patients could be included in the final analysis. **Figure 1** depicts a representative example for the scanned TMAs stained with antibodies and the corresponding overlays generated by QuPath. Good staining quality was achieved, which allowed for the use of automated evaluation, after manually adapting the cut-off value for positive cell counts to the different staining intensity of each antibody.

### Univariable analysis

EORTC intermediate risk group was significantly associated with a shorter RFS compared to the low-risk group (Hazard Ration (HR): 2.08, p = 0.03). However, no statistically significant effect of EORTC high risk classification on RFS was detected (HR 1.18, p = 0.61). Additionally, the EORTC classification system was not predictive for PFS in our cohort. When analyzed individually, higher densities of each of the mIS components (CD3, CD8, CD45RO and FoxP3) were not associated with a longer PFS or RFS. However, when analyzed cumulatively, higher mIS was significantly associated with longer RFS (HR: 0.95, p = 0.04) but not with longer PFS (**Table 2**).

**Table 2:** Univariable cox regression analysis.

Variable	RFS.HR	p-value	PFS.HR	p-value
**EORTC low risk**	1		1	
**EORTC medium risk**	2.08	**0.03**	6.13	0.08
**EORTC high risk**	1.18	0.61	4.63	0.14
**CD3 (n. continuous)**	0.90	0.13	0.76	0.06
**CD8 (n. continuous)**	0.87	0.05	0.84	0.20
**CD45Ro (n. continuous)**	0.93	0.22	1.01	0.95
**FOXP3 (n. continuous)**	0.89	0.12	0.83	0.22
**mIS (n. continuous)**	0.95	**0.03**	0.93	0.15
**Age**	1.03	**0.01**	1.06	**0.01**
**Multifocality**	1.65	0.03	1.76	0.19

RFS, Recurrence free survival; PFS, Progression free survival; HR, Hazard ratio; mIS, modified Immunoscore (CD3, CD8, CD45Ro, FOXP3); EORTC, European Organisation for Research and Treatment of Cancer.Bold values mark statistically significant p values.

### Multivariable analysis

For the full model we combined EORTC risk groups with all four mIS components. Similar to univariable analysis EORTC intermediate risk group was associated with shorter RFS compared to the low risk group (HR 2.04, p=0.04) (**Table 3**). None of the individual mIS components were associated with longer PFS or RFS. When integrating mIS into the EORTC-only model, the concordance index of PFS increased considerably (0.61 to 0.70), but only marginally for RFS (0.57 to 0.60) (**Table 4**).

**Table 3:** Multivariable cox regression analysis.

Variable	RFS.HR	p-value	PFS.HR	p-value
**EORTC low risk**	1		1	
**EORTC medium risk**	2.04	**0.04**	7.23	0.06
**EORTC high risk**	1.02	0.95	3.87	0.20
**CD3**	0.94	0.50	0.72	0.06
**CD8**	0.87	0.11	0.93	0.68
**CD45Ro**	1.08	0.36	1.33	0.10
**FOXP3**	0.89	0.23	0.78	0.18

RFS, Recurrence free survival; PFS, Progression free survival; HR, Hazard ratio; mIS, modified Immunoscore (CD3, CD8, CD45Ro, FOXP3); EORTC, European Organisation for Research and Treatment of Cancer.Bold values mark statistically significant p values.

**Table 4:** Comparison of concordance index.

Outcome	EORTC only model	EORTC only model plus mIS
RFS	0.57	0.60
PFS	0.61	0.70

RFS, Recurrence free survival; PFS, Progression free survival; HR, Hazard ratio; mIS, modified Immunoscore (CD3, CD8, CD45Ro, FOXP3); EORTC, European Organisation for Research and Treatment of Cancer

### Subgroup-analysis according to different EORTC risk groups

**Figure 2:**
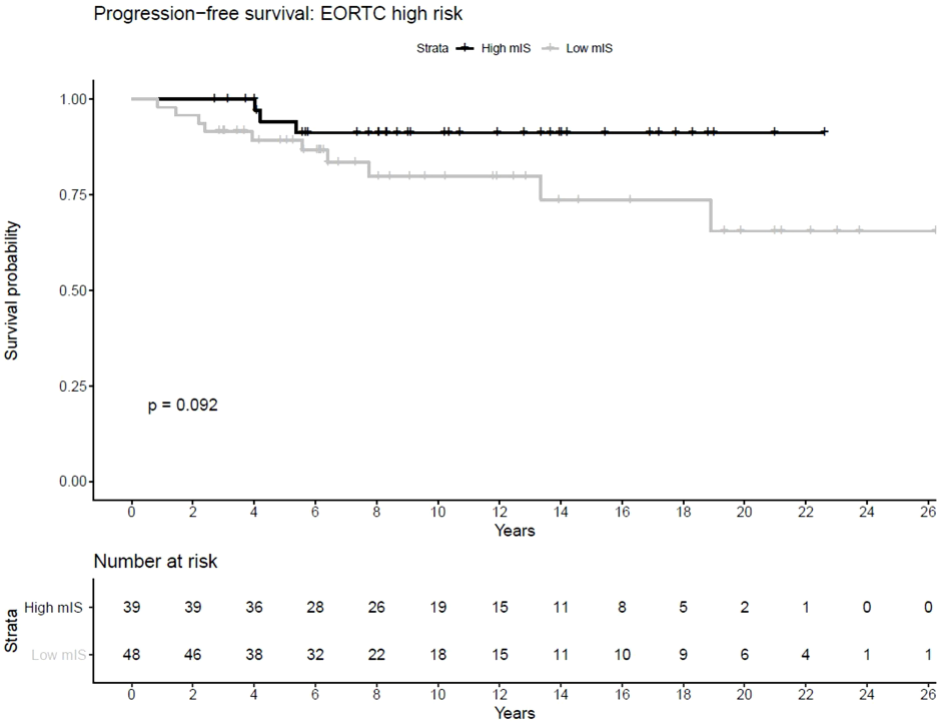
shows the Kaplan Meier curves concerning PFS of low and high mIS patients, demonstrating that patients with high mIS showed a longer PFS in comparison to patients with low mIS.

EORTC high risk patients with low mIS showed a considerably shorter PFS compared to those with high mIS (HR 2.9, Confidence Interval 0.79 – 11.0, p-value (log-rank) = 0.082) (**Table 5**). This observation was confirmed by KM-analysis indicating a distinct division of the survival-curve but without reaching statistical significance (p-value (log-rank) = 0.092), (**Figure 2**). No significant or relevant difference concerning PFS between low and high mIS in EORTC intermediate and low risk groups could be observed. For RFS, no association with mIS in all EORTC risk groups was detected (**Table 5**).

**Table 5:** EORTC-Substratification.

Variable	RFS.H R	CI	p-value	PFS:HR	CI	p-value
**EORTC low risk + high mIS**	1			**1**		
**EORTC low risk + low mIS**	1.3	0.42-4.2	0.64	-	-	–
**EORTC medium risk + high mIS**	1			1		
**EORTC medium risk + low mIS**	1.4	0.72-2.9	0.30	1.6	0.44-6.1	0.46
**EORTC high risk + high mIS**	1			1		
**EORTC high risk + low mIS**	1.4	0.74-2.9	0.30	2.9	0.79–11.0	0.08

RFS, Recurrence free survival; PFS, Progression free survival; HR, Hazard ratio; CI, Confidence interval; mIS, modified Immunoscore (CD3, CD8, CD45Ro, FOXP3); EORTC, European Organisation for Research and Treatment of Cancer.

Because of low patient count within EORTC low risk group and only one patient with progression no substratification in high and low mIS was feasible. Bold values mark statistically significant p values.

## Discussion

This is one of the largest cohorts investigating TIL in NMIBC. The presented data indicates the feasibility of quantitative analyses of TIL in TMAs as to further evaluate TIL and mIS as a possible tool to improve prognosis prediction in NMIBC.

According to our study, mIS seems to increase accuracy in PFS prediction for NMIBC patients. This was most apparent in the EORTC high risk group, where it was possible to substratify patients into a high mIS group with favourable prognosis and a low mIS group with less favourable prognosis.

Since the EORTC classification system is least reliable in high risk situations (10), our findings seem particularly pertinent in this setting. Contrary, mIS was not useful to predict PFS in low or intermediate risk groups. One possible explanation might be that low- to intermediate NMIBC does not trigger a similar strong immune response as compared to high-risk NMIBC. In high-risk cancer a shift towards a more aggressive biology with higher mutational load can be more frequently observed, resulting in increasing neoantigen production. Only a minority of neoantigens are immunogenic, therefore a certain amount of mutations is needed to trigger an immune system reaction against the tumour cells (34–36). Higher TIL densities could be a sign of increased tumour immunogenicity, thus stochastically giving the immune system more opportunities to detect the neoantigen expressing cancer cells and thereby delay cancer progression (20, 37).

Contrary, mIS seems to add very limited information in predicting RFS in NMIBC in comparison to the already established EORTC risk classification. NMIBC recurrence is believed to be mostly dependent on the malignant potential of the entire urothelium and on other factors, like adjuvant treatment status or resection quality (38).

Interestingly, our study published on the value of mIS in MIBC showed that a higher mIS correlated to a longer PFS and CSS in American Joint Committee on Cancer stage IIIa tumours, further hinting that TIL densities and cancer progression are closely related (28).

Once CD4, CD8, FOXP3 and CD45Ro densities were integrated into a combined model, higher densities of all four studied cell types appeared to be significantly associated with prolonged RFS. Concerning PFS, the same can be reported for CD4, CD8 and FOXP3, whereas higher CD45Ro densities seem to have the opposite effect.

A growing number of studies support our findings regarding the prognostic value of CD3 and CD8 positive TIL. Sharma et al. (25) showed in a cohort of MIBC patients that a higher density of intratumoural CD8 cells was linked to longer overall- and disease-free-survival and Nassif et al. (27) found that a higher IS, consisting of CD3 and CD8 densities, translates to prolonged OS and RFS in MIBC. In another study on localised MIBC Nassif et al. (39) were even able to demonstrate that a higher IS translated to a higher rate of complete pathological response after neoadjuvant chemotherapy. Similarly, Horn et al. (12) found that high CD3 and CD8 counts in MIBC were associated with longer OS and CSS. Additionally, in their recent study on MIBC Peng et al. (26) associated higher CD8 densities with longer OS. Similarly, in our recent study we found a longer CSS to be linked to higher CD3 and CD8 densities (28). Contrary, Krpina et al. (40) linked higher CD3 and CD8 counts with an increased risk of cancer recurrence in solitary papillary low grade NMIBC using TMAs. And Hülsen et al. (41) concluded that higher immune cell densities, particularly of CD8, in the invasive tumour margin, correlated with shorter OS in paraffin embedded TURB samples of pT1 tumours. With the available literature a consensus on the impact of TIL on bladder cancer prognosis has yet to be reached. These conflicting results could be due to the analysis of different tumour stages or different sample location within the tumour as well as from analysis of different material, ranging from TMAs over formalin fixed paraffin embedded tissue samples from TURB to whole cystectomy specimen. In comparison to the studies of Hülsen et al. (41) and Krpina et al. (40) our cohort is more diverse, consisting of all non-muscle-invasive tumour stages, histological grades and growth patterns.

FOXP3 is a known reliable marker for Tregs (42). In our study we could only show a non-significant trend towards increased PFS and RFS with higher FOXP3 densities. Winerdal et al. (43) demonstrated, that FOXP3 expressing lymphocytes detected in BC tissue were associated with prolonged survival. However, there are the findings of Horn et al. (12), whereas high FOXP3/CD8 and FOXP3/CD3 ratios were associated with shorter OS and CSS in MIBC. Furthermore, Parodi et al. (44) were able to link a lower T-effector-cells/Treg ratio with BC patients who experienced tumour recurrence. In addition, Loskog et al. (45) found in their study, that intratumoural FOXP3-positive Tregs were involved in creating an immunosuppressive environment in BC. Winerdal et al. (43) suggested that the Tregs detected in their study are in fact not true Tregs but activated CD4+ T-cells who upregulated FOXP3. This finding could offer a possible explanation for the trend observed in our study.

We are aware that our study has certain limitations. The first being sample size, despite having one of the largest cohorts of NMIBC patients examined for TIL up to date. Second, the use of TMAs as opposed to whole tumour slides could be grounds for a potential bias, as the sample cores were taken at random and not from standardised locations within the tumour. However, Peng et al. (26) extrapolated TIL densities by analysing genetic tumour data sets. They were able to associate different cell densities to different outcomes whilst having no information about cell distribution within the tumour. Third, we were unable to assess our dataset concerning the efficacy of mIS in predicting response to adjuvant intravesical BCG-therapy or chemotherapy due to limited data availability in a cohort in which sampling started more than thirty years ago. Despite these limitations, we were able to demonstrate that determination of mIS in TMAs is feasible.

To conclude, our results suggest that quantitative TIL signatures are of prognostic value in NMIBC. mIS holds promise to support further differentiating EORTC high risk patients into a high mIS subgroup with favourable prognosis and a low mIS subgroup with poor prognosis. If confirmed in further studies, mIS could be used in addition to established prognostic factors to allocate high risk NMIBC patients to more streamlined follow-up or more aggressive treatment regimens.

## Data availability statement

The raw data supporting the conclusions of this article will be made available by the authors, without undue reservation.

## Author contributions

Conceptualization, UB and CP. Methodology, UB, LB and MW. Software, LB. Validation, CP. Formal analysis, MW. Resources, HM and DEb. Data curation, DEn. Writing—original draft preparation, DEn. Writing—review and editing, UB, LB, MW, HM, DEb, TH and CP. Supervision, CP. All authors contributed to the article and approved the submitted version.

## Conflict of interest

The authors declare that the research was conducted in the absence of any commercial or financial relationships that could be construed as potential conflict of interest.

## Publisher’s note

All claims expressed in this article are solely those of the authors and do not necessarily represent those of their affiliated organizations, or those of the publisher, the editors and the reviewers. Any product that may be evaluated in this article, or claim that may be made by its manufacturer, is not guaranteed or endorsed by the publisher.
